# The efficiency of sensory systems in postural control of children with and without hearing or visual impairments

**DOI:** 10.1371/journal.pone.0321065

**Published:** 2025-05-12

**Authors:** Hamed Zarei, Ali Asghar Norasteh, Lauren J. Lieberman, Michael W. Ertel, Ali Brian

**Affiliations:** 1 Physical Education & Sport Sciences, (Corrective Exercise and Sport Injuries), Corrective Exercises and Sports Injury Department, Faculty of Physical Education & Sport Sciences, College of Physical Education& sport sciences, University of Guilan, Rasht, Iran; 2 Physiotherapy Department, Faculty of Medicine, Guilan University of Medical Sciences, Rasht, Iran; 3 Department of Kinesiology, Sport Studies and Physical Education, State University of New York (SUNY), Brockport, New York, United States of America; 4 Department of Educational and Developmental Science, University of South Carolina, Columbia, South Carolina, United States of America; 5 Department of Physical Education, University of South Carolina, Columbia, South Carolina, United States of America; UFPE: Universidade Federal de Pernambuco, BRAZIL

## Abstract

Limited evidence exists on the efficiency of the sensory systems of children with sensory impairment. The purpose of this study was to examine the sensory systems involved in postural control of male children with hearing (HI) or visual impairments (VI) compared to those without HI or VI. Participants aged 9–13 years old (*N* = 45, *M*_age _= 11.43, SD = 1.5) were placed within one of three equally stratified purposive groups (HI, VI, comparison children). Postural control was measured using a Kistler force plate (with stabilometric parameters: mean velocity in the anterior-posterior (AP) and medial-lateral (ML), standard deviation (SD) velocity in the AP and ML directions, and sway area) applying four different sensory conditions; condition A: standing on two legs on a stable surface with the eyes open and without any sensory interference (assessment of postural control); condition B: standing on two legs on a stable surface with eyes closed and hyper-extension of the head (perturbation of vestibular and visual system: assessment of proprioceptive system); condition C: standing on two legs on an unstable surface with hyper-extension of the head (perturbation of vestibular and proprioception system: assessment of visual system); condition D: standing on two feet on an unstable surface and eyes closed (perturbation of proprioception and visual system: assessment of vestibular system). The results indicated that in the assessment of postural control condition, comparison children had smaller center of pressure (COP) parameters than children with VI and HI (*p* = 0.001). Also, children with HI had smaller COP parameters compared to VI (*p* = 0.001). In the assessment of proprioceptive system condition, comparison children had greater COP parameters than children with HI and VI (*p* = 0.001). In conclusion, comparison children had better postural control than children with VI and HI; and children with HI had better postural control than children with VI. The proprioceptive system of children with VI and HI performed better than comparison children in maintaining postural control. Consequently, children with VI, HI, and comparison children depend on the proprioceptive system more than other sensory systems to maintain postural control.

## Introduction

Clinically, optimal postural control requires the coordinated integration of information from the visual, vestibular, and proprioceptive systems, all of which must work simultaneously and harmoniously to ensure effective corrective postural control responses [1,2]. Generally, if one of these systems provides incorrect or insufficient information, the remaining intact sensory systems must reweight and deliver accurate and sufficient information to maintain postural control [3,4].

In this regard, the researcher reviewed literature to see how the remaining sensory systems in children with hearing and visual impairment are compared to peers without sensory impairments (Comparison children). There is emerging evidence demonstrating that children with hearing impairment (HI) compensate for the lack of information in the vestibular system through the visual and proprioception systems to maintain static balance with open or closed eyes [5–8]. Additionally, children with HI often will have to maintain postural control while standing when both visual and somatosensory information are available. However, when visual and somatosensory information is perturbed, children with HI have difficulty maintaining their postural control [9–11].

Contradictory evidence has been amassed regarding the predominance of each of these sensory systems in children with HI. Oftentimes, the visual system is predominant in children with HI [9]. An escalating number of studies have reported that the proprioception system may be more responsible for the postural control of children with HI than the visual system [8,12–14]. Given this contradiction in the literature, further investigation is required.

In addition, investigations have also examined balance control in children with visual impairment (VI), revealing that they have poorer balance control compared to their peers without visual impairment [15–19]. There have also been various reports about the efficiency of the sensory systems of children with VI. In closed-eye conditions, children without VI show a postural control level akin to children with VI [20–23]. This finding may suggest that the remaining sensory systems (vestibular and proprioceptive) in children with VI function more effectively in maintaining postural control compared to their peers without sensory disabilities.

Additionally, the balance control of children without VI does not necessarily decrease under the influence of perturbation of the vestibular system [24,25]. The greatest effect was observed during the perturbation of the proprioception system. However, amongst children with VI, balance decrements tend to be more profound when the proprioception system and also the vestibular systems are perturbed [24]. Thus, in the absence of visual information in VI children, children rely more heavily on vestibular and proprioception information [20–22]. However, the proficiency of the proprioception system was expected to be higher in children with VI than in children without VI, which was mentioned only in one study [26]. Therefore, further studies are needed that include the measurement of individual sensory systems to determine whether the proprioceptive and vestibular systems of children with VI function similarly to those of children without VI or HI.

A review of previous literature revealed the efficiency of each of the sensory systems in the postural control of children with HI and VI changes [9,12,13,20–22,27]. Despite this evidence, some studies report contradictory results, which may be attributed to differences in participant ages and the various tools used to measure the efficiency of the sensory systems involved in postural control. Previous studies have typically perturbed only one sensory system at a time, which limits the accurate evaluation of the contributions of the remaining two systems in maintaining postural control. In contrast, the present study utilized a force plate device to measure postural control that perturbs two sensory systems while evaluating the third, allowing for a more accurate assessment.

Peterka [28] investigated the amount of re-weighting of sensory systems information during perturbations in different sensory systems. For individuals without a disability, with perturbation in one of the sensory systems, the amplitude of the postural sway increases immediately. However, with increasing reliance on other sensory systems, the amplitude of the postural sway decreases. Sensory systems information is reweighted in individuals without a disability. However, conflicting results were observed in individuals with vestibular system defects. Sensory system perturbation for individuals with vestibular system defects caused the amplitude of postural sway to increase. However, subsequent sensory reweighting did not occur, and the postural control system was unable to determine which sensory system was providing accurate information to rely more heavily on. Therefore, the amplitude of the postural sway did not decrease. Re-weighting refers to the potential importance of having inertia (gravity) and spatial awareness provided by the vestibular system [29]. Current evidence suggests that it is more effective to perturb two sensory systems and evaluate the role of the remaining one to assess postural control. This approach provides more accurate information about the contributions of the sensory systems in these groups.

To the best of our knowledge, no comprehensive research has yet been conducted to compare the efficiency of sensory systems in children with HI and HI to their peers without sensory impairments or to determine how the absence of one sensory system affects the function of other sensory systems. Therefore, the purpose of this study was to investigate the efficiency of the sensory systems involved in postural control of children with HI and VI compared to comparison children.

## Method

### Participants

Forty-five participants were divided into three equally stratified purposive groups (HI: *n* = 15, VI: *n* = 15, Comparison children: *n* = 15). The participants were within the age range of 9–13 years (*M*_*age*_ = 11.43, *SD* = 1.5) (Table 1). The selection of participants included a standardization based on height, body mass, and age to minimize the confounding impact of these variables on the results of the study.

**Table 1:** Children characteristics.

Characteristic		VI (n = 15)			HI (n = 15)			CP (n = 15)			Total group (n = 45)			p-value
		mean	±	SD	mean	±	SD	mean	±	SD	mean	±	SD	
Age	(y)	11.5	±	2.1	11.9	±	0.9	10.9	±	1.5	11.43	±	1.5	0.41
Height	(cm)	138.7	±	5.4	135.8	±	5.2	136.8	±	5.8	137.1	±	5.4	0.35
Body mass	(kg)	35.2	±	6.2	36.2	±	5.4	37.9	±	5.4	36.4	±	5.6	0.67
Leg length	(cm)	71.36	±	4.3	69.95	±	4.6	70.39	±	4.9	70.56	±	4.6	0.59
BMI	(kg/m^2^)	18.3	±	2.4	19.6	±	2.1	20.2	±	2.1	19.3	±	2.2	0.29

BMI: Body mass index; VI: Visually-impaired; HI: Hearing-impaired; CP: Comparison peers; *significant differences between variables (p ≤ 0.05).

The criteria for the present study included 1) male children (due to environmental and cultural conditions, female participants were not available); 2) Sensorineural hearing loss (the used criterion just for HI children); 3) Moderate to severe VI: Visual loss was classified by the World Health Organization categories of VI. where blindness was defined as best- corrected visual acuity (VA) <3/60 in the better eye, severe VI as VA < 6/60–3/60 in the better eye, and moderate VI as best- corrected visual acuity <6/18–6/60 in the better eye (the used criterion only for VI children); 4) Age of the participants (age range from 9 to 13 years); 5) Hearing range of higher than 75 decibels for hearing impaired children; 6) Not participating in any professional and recreational sports, and only participating in the general physical education classes of the school; 7) No use of psychiatric medications or medications affecting postural control; 8) No hearing loss in VI children and no visual impairment in HI children; 9) Lack of postural abnormalities in the research process (in the lower and upper limbs); and 10) Qualified for physical activity readiness questionnaire and the participants’ health questionnaire.

The exclusion criteria for participants were 1) Rheumatoid arthritis; 2) history of anesthesia during the last 6 months; 3) insulin-dependent diabetes; 4) taking medications affecting the nervous system; 5) history of ankle injury and sprain or knee and hip surgery during the last 6 months; 6) visual and hearing impairment. For comparison children: 1) did not possess any vision correction or cochlear implantation) and 2) brain, vascular disease, or any peripheral and central disease that may be involved in sensory input.

An power analysis was conducted to determine the sufficient sample size of each group. The sample size was calculated based on a previous study by Negahban and Nassadj [30] with an alpha level of 0.05, and an actual power (1-beta) of 0.80. The analysis (G × Power, Version 3.1.9.2, University of Kiel, Germany) revealed that a sample size of *n* = 15 would be sufficient for each group to find significant effects between variables

### Study design

The start and end of the recruitment period for this study was 15/9/2023 and 16/12/2023 respectively. This study featured a descriptive/analytic and cross-sectional design. children with HI from a regional deaf center within a local deaf school, children with VI from the Center of the Blind Association, and comparison children were also selected from one of the local community schools (public schools) volunteered to participate in the study. Children were pair-matched according to their sensory disorders. After studying the medical records of HI participants, those with complete hearing loss (of the sensorineural type) (hearing range greater than 75 decibels) were selected for the study, and for the VI children, the range of moderate to severe visual impairment was selected. Two days before administering the tests, a thorough explanation of the study was given to the participants and parents of children; and parents’ consent was obtained for their children’s participation in this research. The height and body mass of the participants were measured before starting the tests of postural control. Also, their body mass index was calculated. Then, to start the test, the actual leg length, i.e., from the anterior superior iliac spine to the medial malleolus of the ankle, was measured to normalize the data and compare the participants without worrying about individual differences [31]. Data collection was performed across 3 successive days including; day 1) Measuring tests of HI children; day 2) Measuring tests of VI children; and 3) Measuring tests of comparison children. Each participant was supervised and verbally guided to achieve proper implementation of each test by a researcher and physiotherapist to control all the testing procedures. all testing were conducted by both a physiotherapist and a researcher who had sufficient skills and specialized certificates in the field of these testing with force plate.

**Fig 1:**
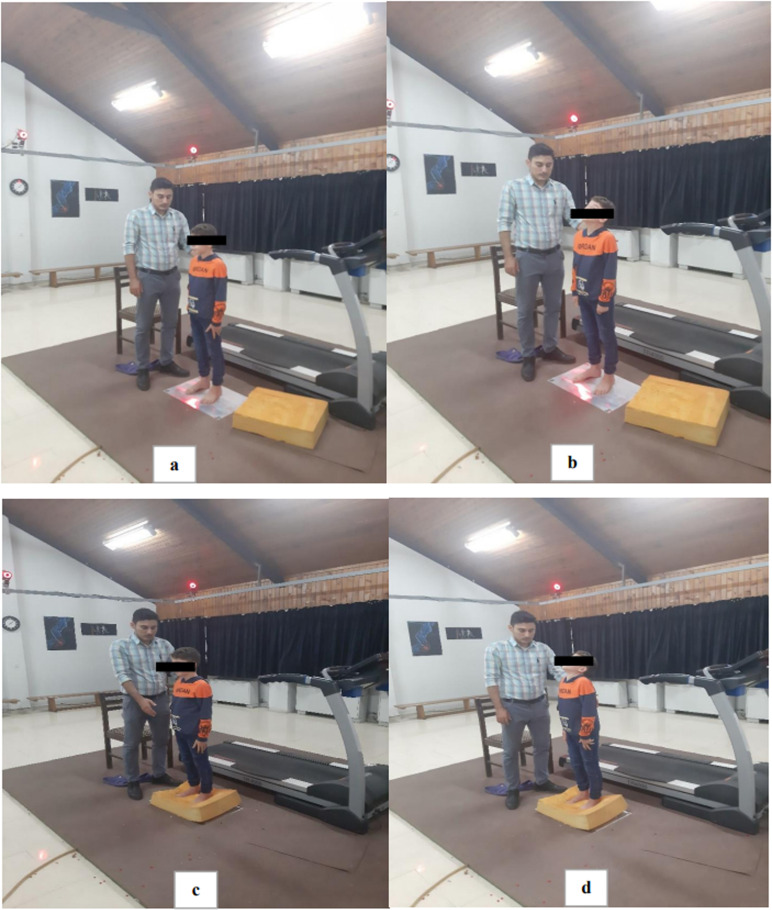
Evaluation of postural control under four different sensory conditions : condition A, standing on two legs on a stable surface with the eyes open and without any sensory interference (assessment of postural control); condition B, standing on two legs on a stable surface with eyes closed and hyper-extension of the head (assessment of proprioception system); condition C, standing on two legs on an unstable surface with hyper-extension of the head (assessment of visual system); condition D, standing on two feet on an unstable surface and eyes closed (assessment of vestibular system).

Before the administration of the measurements, the Medical and Sports Information Questionnaire was applied. These questionnaires provided information about cardiovascular, respiratory problems, physical health, and were a screening tool so that children who did not have specific health and physical problems were included in the study [32]. Then, parents’ written informed consent was collected for both study participation and publication of identifying information/images in an online open-access publication. Participants were informed about the nature of the study and they were assured that the measurement methods are not dangerous. This trial obtained ethical approval from the Institute of Sport Sciences Research Institute (SSRI) IR. SSRI. REC. 1399. 028; and all experiments were performed in accordance with relevant guidelines and regulations. The legal guardians of the individual pictured in Fig 1 has provided written informed consent (as outlined in PLOS consent form) to publish their image alongside the manuscript. Furthermore, the individual pictured in Fig 1 has provided written informed consent (as outlined in PLOS consent form) to publish their image alongside the manuscript.

### Measurements

Height was measured by a stadiometer (Seca 222, Terre Haute, IN) mounted on the wall and recorded to the nearest 0.5 cm [33]. Body mass was measured to the nearest 0.1 kg using a digital scale (Tanita, BC-418MA, Tokyo, Japan) [33]. Body mass index (BMI) was calculated (kg/m^2^).

Postural control was measured by a Kistler force plate model BA 9286 (made in Switzerland) using four different sensory conditions with a frequency of 1000 Hz and second-order low-pass Butterworth filter frequency of 20 Hz. [30].

### Procedure

Center of pressure (COP) sways require the measuring of the postural sway indices of mean velocity in the anterior-posterior (AP) and medial-lateral (ML), standard deviation (SD) velocity in the AP and ML directions, and sway area (95% confidence ellipse). Demonstrated reliability from previous research guided the use of mean velocity and standard deviation of velocity. [34,35]. Also, the sway area was chosen to increase the accuracy of the measurements [36]. Displacement, mean and velocity of the COP sways were calculated by Excel software. To eliminate the potential noise, all data were filtered using a second-order low-pass Butterworth filter [30]. A comparison of the COP sway area components in four sensory conditions and between different groups was performed in the Matlab software R2024b (The MathWorks, Inc., Natick, Massachusetts, United States) [30].The efficiency of sensory systems in postural control was measured in four different sensory conditions, including: condition A, standing on two legs on a stable surface with the eyes open and without any sensory interference (assessment of postural control); condition B, standing on two legs on a stable surface with eyes closed and hyper-extension of the head (perturbation of vestibular and visual system: assessment of proprioceptive system); condition C, standing on two legs on an unstable surface with hyper-extension of the head (perturbation of vestibular and proprioception system: assessment of visual system); condition D, standing on two feet on an unstable surface and eyes closed (perturbation of proprioception and visual system: assessment of vestibular system) (Fig 1).

Four different sensory conditions that were used to evaluate the efficiency of the sensory systems of postural control were similar to the conditions of the sensory organization test (SOT). In the SOT, there were six conditions and, in each condition, only one sensory system information was disturbed, and two sensory systems information were evaluated. But, in the test used in the present study, there were four conditions, two of which were removed compared to the SOT. Two sensory system information were disturbed in each condition, and only one sensory system information was measured. Therefore, there is no integration of sensory systems when evaluating each condition; and to evaluate better information from each of the sensory systems information. SOT is a form of posturography that is designed to assess quantitatively an individual`s ability to use visual, proprioceptive, and vestibular cues to maintain postural stability in stance [37]. The SOT conditions significantly influence the postural strategy adopted, where the availability and unavailability of accurate and/or conflicting sensory feedback, based on the type of SOT condition, can influence the study findings [38]. Changes in the vertical position of the head may induce instability by placing the utricular otolith organs beyond their working range [39]. Vertical position changes may explain why participants demonstrate increased standing postural instability with their heads extended.

In the foam conditions, participants were instructed to stand on a foam pad (40 × 60 cm dimensions, 10 cm thickness, and 35 kg/m3 density) placed on the forced platform. Placing a foam pad on a force plate does not significantly alter the force readings, as demonstrated by Patel, Fransson [40] who found that the addition of a foam layer did not impact the accuracy of force measurements in their experiments. In each position, the participants’ hands were against their waist. The participants stood with their feet positioned shoulder-width apart, ensuring a standardized stance across all participants. The distance between the feet was approximately 15–20 centimeters, consistent regardless of the children’s height. All participants were barefoot or wearing stockinged during the assessment, providing a uniform condition for the experiment. All test conditions were explained to each subject via total communication, body language, and sign language involving speech and demonstration. The instructions that were given to the participants when they were placed on the force plate included: 1) stand on the force plate as still as they could and face forward on a point that is two meters away from the force plate on the wall; 2) During the disturbance in the vestibular system: raise your head as far as you can and look at the ceiling. 3) Do not remove your hands from your waist at all. 4) During disturbances in the visual system: close your eyes and do not open them at all until you are instruction to relax. If participants committed any of the specified errors, the trial was promptly stopped and repeated to maintain uniformity in the testing conditions. Each participant performed the test for 30 seconds [41]. Each condition was repeated in 3 trials lasting for 30 s (each trial). The presentation of all postural conditions (A, B, C, D) was counterbalanced for each subject to minimize learning effects. However, the three trials of each postural condition were completed sequentially. Moreover, a break time of five minutes was considered between postural conditions to minimize fatigue. The participants were asked to sit on a chair during the break time.

### Statistical analysis

The Shapiro-Wilk Normality test was used to check the normal distribution of data. All the values are presented as mean ± standard deviation (SD). To check the differences between children’s characteristics, the primary analysis was an independent samples *t*-test. A repeated-measures ANOVA (3 groups × 4 conditions) was utilized to analyze data (SPSS 28.0) [42]. Assumptions of sphericity were assessed using Mauchly’s test of sphericity, with any violations adjusted by using the Greenhouse-Geisser (GG) correction. When a significant F value was achieved, a Scheffé post hoc test was used to detect differences in the measures [42]. A difference was statistically significant at *p* ≤ 0.05. Cohen’s d [43] was also used to calculate the effects of group and condition (effect size). Threshold values for assessing magnitudes of ES were <0.2, trivial; 0.2–0.6, small; 0.6–1.2, moderate; 1.2–2.0, large; 2.0–4.0, very large; and > 4.0, nearly perfect [44]. The effect size is reported with a 95% confidence interval (CI) for all analyzed measures.

## Results

There were no anthropometric differences between the three groups for age (*p* = 0.41), height (*p* = 0.35), body mass (*p* = 0.67), leg length (*p* = 0.59), and BMI (*p* = 0.29). The results of the repeated-measures ANOVA showed that the interaction of the group by postural conditions was significant for mean velocity in AP (*F* = 6.78, *p* = 0.001), mean velocity in ML (*F* = 7.36, *p* = 0.001), SD velocity in AP (*F* = 8.39, *p* = 0.001), SD velocity in ML (*F* = 7.36, *p* = 0.001) and Sway area (*F* = 9.16, *p* = 0.001) (Tables 2 and 3). Scheffé’s post hoc test was used to detect differences in the measures.

**Table 2:** Mean ± SD of COP parameters in different postural conditions in the groups.

Groups	Visually-impaired	Hearing-impaired	Comparison peers
Condition A: assessment of postural control			
Mean Velocity, AP	23.61 ± 19.00	21.97 ± 7.46	15.37 ± 6.92
Mean velocity, ML	16.44 ± 9.45	14.60 ± 6.40	12.33 ± 5.84
SD Velocity, AP	40.14 ± 52.43	25.35 ± 10.34	15.44 ± 9.61
SD Velocity, ML	24.11 ± 20.94	18.49 ± 5.89	11.19 ± 5.39
Sway area (95% ellipse)	205.08 ± 233	153.36 ± 338.78	48.26 ± 331.55
Condition B: assessment of proprioceptive system			
Mean Velocity, AP	19.06 ± 6.08	19.47 ± 9.33	22.56 ± 9.17
Mean velocity, ML	13.97 ± 6.03	12.39 ± 4.93	16.03 ± 8.97
SD Velocity, AP	16.89 ± 6.10	23.58 ± 18.67	30.49 ± 58.96
SD Velocity, ML	13.02 ± 5.54	13.94 ± 6.46	24.57 ± 51.87
Sway area (95% ellipse)	91.29 ± 768.77	112.47 ± 103.0	233.88 ± 635.23
Condition C: assessment of visual system			
Mean Velocity, AP	39.65 ± 15.20	36.32 ± 10.72	37.00 ± 11.65
Mean velocity, ML	28.84 ± 16.15	23.86 ± 4.87	25.01 ± 4.48
SD Velocity, AP	43.17 ± 16.41	34.56 ± 12.17	34.03 ± 13.16
SD Velocity, ML	36.83 ± 26.77	22.05 ± 5.87	23.10 ± 5.88
Sway area (95% ellipse)	493.77 ± 745.88	258.12 ± 101.38	273.22 ± 105.87
Condition D: assessment of vestibular system			
Mean Velocity, AP	28.93 ± 6.56	45.47 ± 16.98	29.36 ± 7.12
Mean velocity, ML	23.50 ± 6.67	29.84 ± 12.09	24.41 ± 6.98
SD Velocity, AP	25.30 ± 5.30	47.38 ± 19.68	25.43 ± 5.77
SD Velocity, ML	21.49 ± 6.89	32.25 ± 16.66	22.19 ± 7.24
Sway area (95% ellipse)	295.13 ± 155.25	462.69 ± 337.96	317.64 ± 160.66

COP: center of pressure; SD: standard deviation; AP: anteroposterior; ML: mediolateral. Units of COP parameters are as follows: mm/s (mean velocity and SD velocity); mm^2^ (area).

**Table 3:** Summary of repeated-measures ANOVA for COP parameters: F-ratios and P-values by variable.

		Mean Velocity, AP	Mean velocity, ML	SD Velocity, AP	SD Velocity, ML	Sway area (95% ellipse)
Main effect Condition	F	29.53	19.68	17.98	21.06	32.69
	P	**0.001**	**0.001**	**0.002**	**0.001**	**0.001**
Group	F	6.35	5.98	4.69	6.01	8.69
	P	**0.001**	**0.001**	**0.003**	**0.001**	**0.001**
Interaction Condition × group	F	6.78	7.36	8.39	7.36	9.16
	P	**0.001**	**0.001**	**0.001**	**0.001**	**0.001**

Significant p-values are in bold (p ≤ 0.05).

AP: anteroposterior; ML: mediolateral.

The results of multiple comparisons showed that within the assessment of postural control condition, there were smaller COP parameters in the comparison children (mean velocity in AP, mean velocity in ML, SD velocity in AP, SD velocity in ML and Sway area) than in the HI (*p* = 0.001) and the VI (*p* = 0.001). In addition, there were greater COP parameters in the VI than in the HI (*p* = 0.001).

In the condition assessment of the proprioceptive system, there were greater COP parameters in the comparison children than in the HI (*p* = 0.001) and the VI (*p* = 0.001). Additionally, there was no significant difference in the COP parameters of VI and HI children (*p* ˃ 0.05). In the condition assessment of the visual system, there were greater COP parameters in the VI than in the comparison children (*p* = 0.001) and the HI (*p* = 0.001). There was no significant difference between the COP parameters of HI and comparison children (*p* ˃ 0.05). In the condition assessment of the vestibular system, there were greater COP parameters in the HI than in the comparison children (*p* = 0.001) and the VI (*p* = 0.001). There was no significant difference between the COP parameters of VI and comparison children (*p* ˃ 0.05) (Table 4).

**Table 4:** Summary of multiple comparisons Scheffé’s post hoc test for COP parameters: Effect size (95% CI) by variable.

		Mean Velocity, AP	p-Value	Mean velocity, ML	p-Value	SD Velocity, AP	p-Value	SD Velocity, ML	p-Value	Sway area (95% ellipse)	p-Value
Condition A: assessment of postural control											
	HI-VI	0.06, (-16.25 to -4.34) *	0.04	0.08, (-12.15 to -6.87) *	0.03	0.09, (-13.36 to -8.31) *	0.02	0.09, (-14.46 to -7.36) *	0.02	0.08, (-10.36 to -5.82) *	0.03
	HI-CP	1.1, (7.11 to 12.21) **	0.01	0.09, (5.11 to 12.34) *	0.02	0.08, (6.58 to 13.26) *	0.02	1.5, (7.64 to 11.96) *	0.001	1.9, (5.29 to 11.98) ***	0.001
	VI-CP	1.5, (4.21 to 11.36) ***	0.001	1.3, (8.69 to 18.36) ***	0.001	1.2, (6.25 to 14.34) **	0.01	1.7, (12.36 to 28.36) ***	0.001	2.1, (13.69 to 19.68) ***	0.001
Condition B: assessment of proprioceptive system											
	HI-VI	0.2, (6.18 to 8.37)	0.23	0.1, (6.74 to 9.14)	0.14	0.3, (4.98 to 9.61)	0.35	0.1, (4.69 to 5.99)	0.18	0.3, (2.21 to 3.65)	0.45
	HI-CP	0.9, (2.36 to 5.36) **	0.01	1.2, (4.12 to 8.36) **	0.01	1.1, (3.69 to 7.15) **	0.01	1.2, (4.35 to 10.39) **	0.01	1.2, (6.36 to 11.95) **	0.01
	VI-CP	0.9, (2.98 to 12.36) **	0.01	1.1, (4.36 to 9.68) **	0.01	1.5, (5.38 to 13.69) **	0.01	1.3, (3.68 to 15.96) **	0.01	1.8, (6.36 to 9.68) ***	0.001
Condition C: assessment of visual system											
	HI-VI	1.7, (-6.25 to -4.34) ***	0.001	1.8, (-7.24 to -3.31) ***	0.001	1.9, (-2.38 to 2.95) *	0.001	1.7, (4.95 to 10.35) ***	0.001	1.9, (7.06 to 15.34) ***	0.001
	HI-CP	0.4, (2.17 to 5.87)	0.35	0.3, (9.45 to 12.27)	0.43	0.2, (10.18 to 14.21)	0.29	0.3, (1.58 to 5.74)	0.34	0.06, (2.19 to 4.21)	0.29
	VI-CP	1.6, (6.25 to 11.36) ***	0.001	1.7, (2.19 to 13.68) ***	0.001	1.9, (5.98 to 15.34) *	0.001	1.6, (10.36 to 17.89) ***	0.001	1.8, (1.26 to 4.31) ***	0.001
Condition D: assessment of vestibular system											
	HI-VI	1.7, (5.75 to 11.12) ***	0.001	1.8, (7.38 to 15.94) ***	0.001	1.6, (-18.95 to -14.24) ***	0.001	1.1, (4.48 to 14.98) **	0.01	1.5, (1.25 to 7.68) ***	0.001
	HI-CP	1.6, (-16.35 to -4.31) ***	0.001	1.7, (-15.19 to -11.29) ***	0.001	1.6, (-18.21 to -10.12) ***	0.001	1.2, (-10.15 to -3.84) **	0.01	1.4, (-13.51 to -6.42) **	0.01
	VI-CP	0.2, (7.95 to 12.83)	0.37	0.09, (3.41 to 8.84)	0.37	0.3, (10.25 to 16.89)	0.26	0.2, (6.19 to 10.95)	0.16	0.5, (12.75 to 15.36)	0.34
VI group											
	A-B	1.1, (4.89 to 10.22) *	0.01	1.2, (3.98 to 9.28) **	0.01	1.1, (7.28 to 18.24) **	0.01	1.2, (3.58 to 8.68) **	0.01	1.2, (8.98 to 12.31) *	0.01
	A-C	0.4, (11.21 to 15.13)	0.23	0.6, (-2.97 to 2.36)	0.26	0.5, (6.24 to 10.18)	0.03	0.07, (5.24 to 8.29)	0.03	0.3, (4.47 to 10.28)	0.16
	A-D	0.3, (7.29 to 11.21)	0.33	0.3, (-5.85 to 1.87)	0.24	0.6, (5.49 to 13.28)	0.03	0.3, (-9.21 to -6.14)	0.03	0.4, (-8.36 to -1.27)	0.43
	B-C	1.4, (3.98 to 12.47) **	0.01	1.8, (5.68 to 15.39) ***	0.001	1.3, (2.98 to 7.14) **	0.01	1.4, (4.21 to 14.68) **	0.01	1.7, (7.15 to 12.39) ***	0.001
	B-D	1.6, (1.25 to 4.37) ***	0.001	1.9, (2.29 to 5.38) ***	0.001	1.4, (1.85 to 9.28) **	0.01	1.5, (6.58 to 9.38) ***	0.001	1.8, (7.59 to 13.26) ***	0.001
	C-D	0.08, (4.15 to 8.57)	0.26	0.1, (-7.24 to -3.84)	0.16	0.3, (2.37 to 8.47)	0.03	0.1, (-3.95 to 2.24)	0.26	0.2, (-5.98 to 2.68)	0.27
HI group											
	A-B	1.1, (-6.25 to -1.24) **	0.01	1.2, (-9.25 to -2.37) **	0.01	1.3, (-16.85 to -8.37) **	0.01	1.3, (-6.78 to 1.87) **	0.01	0.9, (-8.21 to -2.37) *	0.02
	A-C	0.4, (-7.14 to -1.84)	0.43	0.4, (-9.12 to -2.27)	0.26	0.3, (-6.28 to -3.65)	0.18	0.4, (-8.24 to -1.38)	0.26	0.3, (-7.13 to -4.27)	0.16
	A-D	0.2, (5.24 to 7.68)	0.19	0.2, (-3.37 to 2.49)	0.34	0.4, (7.23 to 11.74)	0.19	0.2, (5.74 to 9.37)	0.24	0.2, (2.27 to 9.17)	0.18
	B-C	1.7, (2.98 to 9.37) ***	0.001	1.6, (5.51 to 11.21) ***	0.001	1.6, (14.25 to 24.14) ***	0.001	1.8, (18.27 to 24.84) ***	0.001	1.3, (7.22 to 10.31) **	0.001
	B-D	1.8, (9.47 to 17.84) ***	0.001	1.7, (3.21 to 6.38) ***	0.001	1.7, (13.25 to 20.31) ***	0.001	1.8, (15.84 to 23.31) ***	0.001	1.2, (4.21 to 10.48) **	0.001
	C-D	0.4, (1.37 to 6.37)	0.09	0.3, (2.21 to 7.28)	0.18	0.2, (6.12 to 9.28)	0.39	0.2, (5.12 to 10.51)	0.46	0.1, (9.26 to 12.32)	0.24
CP group											
	A-B	0.8, (-11.29 to -4.21) *	0.01	0.7, (-12.54 to -3.24) *	0.02	0.8, (-9.25 to -2.34) *	0.02	0.9, (-8.21 to -4.37) *	0.02	0.7, (-12.28 to -7.84) *	0.02
	A-C	1.3, (6.25 to 14.34) **	0.001	1.9, (3.84 to 10.74) ***	0.001	1.8, (10.25 to 19.84) ***	0.001	1.7, (4.43 to 8.37) ***	0.001	1.5, (13.84 to 19.37) ***	0.001
	A-D	1.5, (15.26 to 27.31) ***	0.001	1.8, (8.29 to 16.78) ***	0.001	1.7, (3.61 to 8.49) ***	0.001	1.6, (2.98 to 7.64) ***	0.001	1.3, (1.84 to 6.48) **	0.01
	B-C	0.7, (-12.38 to -6.24) *	0.02	1.7, (-10.52 to -2.27) ***	0.001	1.6, (-9.32 to -1.48) ***	0.001	1.5, (-16.95 to -9.37) ***	0.001	1.4, (-17.81 to -11.37) **	0.01
	B-D	0.8, (8.21 to 12.37) *	0.02	1.6, (4.21 to 8.37) ***	0.001	1.2, (7.22 to 13.34) **	0.01	1.6, (2.48 to 9.31) *	0.001	1.5, (5.21 to 11.74) ***	0.001
	C-D	0.2, (6.21 to 12.47)	0.09	0.3, (-2.81 to 2.64)	0.28	0.1, (10.38 to 18.24)	0.09	0.2, (-11.78 to -2.37)	0.18	0.3, (4.79 to 12.27)	0.19

AP: anteroposterior; ML: mediolateral; VI: Visually-impaired; HI: Hearing-impaired; CP: Comparison peers; A: Condition A (Assessment of postural control); B: Condition B (Assessment of proprioceptive system); C: Condition C (Assessment of visual system); D: Condition D (Assessment of vestibular system); *significant differences between variables (p ≤ 0.05); **significant differences between variables (p ≤ 0.01); ***significant differences between variables (p ≤ 0.001).

CI: confidence interval.

Within the VI group, there were smaller COP parameters in the condition assessment of the proprioceptive system than in postural control (*p* = 0.001), visual system (*p* = 0.001), and vestibular system (*p* = 0.001). There were no significant differences between the COP parameters of other assessments of sensory/postural conditions (*p* ˃ 0.05). In the HI group, there were smaller COP parameters in the condition assessment of the proprioceptive system postural control (*p* = 0.001), visual system (*p* = 0.001), and vestibular system (*p* = 0.001). Additionally, there were no significant differences between the COP parameters of other assessment of sensory/postural conditions (*p* ˃ 0.05).

In the comparison children group, there were smaller COP parameters in the condition assessment of postural control than in the condition assessment of the proprioceptive system (*p* = 0.001), visual system (*p* = 0.001), and vestibular system (*p* = 0.001). Within the comparison children group, there were smaller COP parameters in the condition assessment of the proprioceptive system than condition assessment of the visual system (*p* = 0.001) and vestibular system (*p* = 0.001). There were no significant differences between the COP parameters of condition assessment of the visual and vestibular system (*p* ˃ 0.05) (Table 4).

## Discussion

To our knowledge, no thorough research has been carried out to compare the efficiency of sensory systems in children with HI and VI compared to comparison children, nor to investigate how the absence of one sensory system influences the functioning of other sensory systems. Therefore, the purpose of this study was to examine the efficiency of sensory systems in postural control of children with HI and VI compared to comparison children. In this regard, postural control was measured in different sensory conditions. In the first condition (condition assessment of postural control), there was no perturbation in the sensory systems. In the second condition (condition B), there was a perturbation in the visual and vestibular systems, and the dominant system for maintaining postural control was proprioception. In the third condition (condition C), perturbation occurred in proprioceptive and vestibular systems, and the dominant system for maintaining postural control was vision. In the fourth condition (condition D), perturbation occurred in the proprioceptive and visual systems, and the dominant system for maintaining postural control was vestibular.

### Postural control

The results of the condition assessment of postural control showed that comparison children had smaller COP parameters than children with VI and HI. Also, in comparison children to children with VI, children with HI had smaller COP parameters. These results revealed that comparison children have better postural control than children with VI and HI; and children with HI have better postural control than children with VI. As vision and vestibular systems are sensory systems for maintaining postural control [45], defects in these systems lead to defects in maintaining postural control. The findings of this study are in line with the results of previously conducted studies that reported postural control defects in children with HI and VI [46,47].

Furthermore, the results demonstrated that those children with HI had better postural control performance than children with VI. The role of the visual system in maintaining postural control is greater than that of the vestibular system [45]. Therefore, defects in the visual system impair postural control more than defects in the vestibular system. The visual system plays a crucial role in providing essential information regarding environmental context and body orientation, whereas the vestibular system is responsible for detecting head position and movement [48]. Research demonstrates that when visual information is accessible, the central nervous system prioritizes this input over vestibular signals in the regulation of balance [3]. This phenomenon is particularly pronounced in scenarios where visual cues are robust or when the environmental conditions are stable [49]. Consequently, it appears that the contribution of the visual system to the maintenance of postural control is significantly greater than that of the vestibular system. Our results show that children with VI have more problems in postural control than children with HI. These results align with previous literature as postural control plays an important role in learning motor skills and increasing physical activity [46,50]. The development of motor skills and participation in physical activity are two essential parts of increasing the quality of life [51,52]. Therefore, a progressive training program should be considered to improve the postural control of children with sensory disorders, especially children with VI.

### Contribution of the proprioceptive system

The comparison children had greater COP parameters than children with HI and VI and there was no significant difference between children with VI and HI in the assessment of proprioceptive system condition. These results showed that the proprioceptive system of children with VI and HI performed better than comparison children in maintaining postural control and there was no significant difference in the proprioceptive system of children with VI and HI. The results of the present study are in line with the obtained results of previous studies [26,53]. They concluded that the proprioceptive system of children with VI is significantly stronger than that of children without VI. However, these results also conflict with the previous studies with no significant differences [20–22]. Research indicates that in children with visual impairment, the visual cortex is often repurposed by other sensory modalities [54]. Complete visual deprivation during early development has been shown to enhance the sensitivity of sensory receptors [55]. Given that the development of spatial cognitive abilities relies on motor skills, it is likely that spatial frames of reference undergo modifications after the first year of life [56]. Thus, the absence of visual feedback during this critical developmental period appears to disrupt the spatial coding necessary for motor control [57]. Therefore, it appears that other remaining sensory systems are enhanced in maintaining postural control.

By removing the visual system. Ribeiro and Olivera (2007) reported that proprioception is considered the most important and main system for the sensory control of balance and helps to stabilize the body and joints through two feedback and feedforward mechanisms [58]. Proprioceptive information is used to regulate overall body balance and joint stability and determines whether the body is moving with the required effort or not, as well as how different parts of the body are positioned relative to each other [59]. Given the critical role of the proprioceptive system in maintaining postural control, children with VI and HI may benefit from proprioceptive training. This training approach may help compensate for postural control deficits resulting from the absence of visual or vestibular sensory input.

### Contribution of the visual system

In the assessment of visual system condition, children with VI had greater COP parameters than children with HI and comparison children. Additionally, no significant differences were found between children with HI and comparison children. In maintaining postural control, the results showed that the visual system of children with VI is weaker than children with HI and comparison children. Also, there was no significant difference between the performance of the visual system among children with HI and comparison children in maintaining postural control. The results of the present study are compatible with the results of previously done studies that showed that the information of the visual system of children with HI works similarly to comparison children in maintaining postural control [12,13,60]. In children with HI, the weakness of postural control caused by vestibular system defects is partially compensated by other sensory systems. Therefore, it seems that performing visual sensory training can improve the postural control of children with HI.

### Contribution of the vestibular system

In the assessment of vestibular system condition, children with HI had greater COP parameters than children with VI and comparison children and there was no significant difference between children with VI and comparison children. The results of the present study exposed that the vestibular system of children with HI was weaker than children with VI and comparison children in maintaining postural control. Also, there was no significant difference found between the vestibular system performance of children with VI and comparison children in maintaining postural control. The results of the present study are aligned with the results of several earlier studies that show the information on the vestibular system of children with VI works like comparison children in maintaining postural control [20,22]. Physiologically, the function of the vestibular system components (including the semicircular canals, otolith organs, and degree of myelination of the vestibular nerve) at birth is similar to that of adults [61,62]. However, the degree of its organization has been reported to be completely different from its physiological function, and studies have shown that even by the age of 15, the organization of the vestibular system is not the same as that of adults [45,63]. Therefore, it is recommended that the implementation of vestibular exercises may improve the function of the vestibular system in children with HI and VI and thus improve postural control in these children [64,65].

### Predominance of the sensory systems in the children with HI and VI

In examining the conditions in each group, the results showed that within the VI group, there were smaller COP parameters in the assessment of proprioceptive system condition than in the other assessment of sensory/postural conditions. Also, among the HI group, the same results as the VI group were observed. Therefore, the results demonstrated that VI and HI groups were dependent on the proprioceptive system to maintain postural control more than other systems. Moreover, in comparison to other sensory systems, the proprioceptive system of these children had a better performance. The results of the present study are in line with the findings of previous studies that reported that the proprioceptive system in the HI group [12,13] and VI group [20,22] is the dominant system for maintaining postural control. The obtained results contradict with some studies that reported children with HI were more dependent on the visual system to maintain postural control [9,27]. The reasons for this contradiction may be the method for calculating the postural control tests and the applied tools. As stated earlier, the vestibular system is critical for visual stabilization (the ability to gaze at something) [66]. Consequently, damage to the vestibular system causes defects in postural control and visual performance. Therefore, the same factor may be the cause of the lower efficiency of the visual system compared to the proprioceptive system in children with HI to maintain postural control.

Also, in a study conducted by Rogge, Hötting [67], the effects of combined proprioceptive-vestibular training on balance and cortical function in individuals with visual impairment were investigated. The findings indicated that after 12 weeks of training, the balance of participants with visual impairment was comparable to that of individuals with typical development. Additionally, there was a notable improvement in the cortical surface thickness of those with visual impairment following the training. These results suggest that such training positively influences the plasticity of the cerebral cortex in individuals with visual impairment [67]. Furthermore, the concept of compensatory plasticity within the reciprocal model remains applicable to this population. Consequently, it appears that children with HI and VI, in the absence of one sensory system, tend to rely more heavily on their remaining sensory modalities, particularly proprioception, for daily activities and social interactions [68]. This increased dependence on proprioceptive feedback during everyday tasks may further enhance the functionality and strength of the proprioceptive system.

### Predominance of the sensory systems in the comparison children

Multiple comparisons showed that within the comparison children group, there were smaller COP parameters in the assessment of postural control condition than in the other assessment of sensory/postural conditions. Smaller COP parameters also existed in the assessment of proprioceptive system condition than in the assessment of visual and vestibular system condition. The results revealed that, like children with VI and HI, comparison children depend more on the proprioceptive system than other sensory systems to maintain postural control. The proprioceptive system of comparison children performed better than other systems to maintain postural control. However, unlike children with HI and VI, comparison children had better balance performance in the assessment of postural control condition than in other assessments of sensory/postural conditions. These results displayed that the reweighting of sensory systems information in children with VI and HI develops earlier than comparison children. Also, the integration of sensory systems information in comparison to children occurs earlier than in children with VI and HI. Therefore, it seems that the implementation of training programs that improve the remaining sensory systems in children with HI and VI may have a significant effect on the postural control of these children.

## Strength and limitations

The current study had several strengths. To the best knowledge of the researchers, this was the first to investigate the efficiency of sensory systems in three groups of children with HI, VI, and comparison children, while previous studies have been conducted between two groups. The comparison of children of the three groups provides more accurate and complete information about the efficiency of the three sensory systems (visual, vestibular, and proprioception) in maintaining postural control. The instrument used to assess postural control was psychometrically robust, whereas previous studies had used more functional instruments. Also, the method of evaluating sensory systems was innovative. In this study, two sensory systems were disturbed and one sensory system was evaluated. In previous studies, only one sensory system was disturbed, which caused a high probability of error and a lack of accurate assessment of the sensory systems.

This study had limitations to report. Due to the limited number of participants, the findings of the present study are only generalizable to 9–13-year-old male children. More research is needed to identify whether our findings can be generalized to female children or a different age band. Also, in the current study, visual, auditory, and vestibular examinations to assess the children’s levels of disability were not conducted. Therefore, it is strongly recommended that future research incorporates these assessments. Finally, the present study just evaluates static postural control. Further studies can be conducted on the dynamic balance and motor skills of these people to determine the differences between the three groups in these variables.

## Conclusion

In summary, this study found that children without a disability exhibit superior postural control compared to those with visual VI and HI, with HI children demonstrating better postural control than VI children. Additionally, the proprioceptive system in children with VI and HI performed better in maintaining postural control than in comparison children. The results also indicated that all groups—children with VI, HI, and comparison children—relied more on their proprioceptive system than on other sensory systems for postural control. Proprioceptive training tailored to the strengths of the sensory systems in children with HI and VI could enhance their postural control. However, in the absence of sensory disturbances, comparison children showed better balance performance across other sensory and postural assessments compared to those with HI and VI. These findings suggest that sensory system reweighting develops earlier in children with VI and HI compared to comparison children, who integrate sensory systems more effectively.

## Supporting information

S1 File Inclusivity-in-global-research-questionnaire. (DOCX)

S2 File Minimal data set. (SAV)

## References

[1] Ivanenko Y, Gurfinkel VS. Human postural control. Front Neurosci. 2018;12:171. doi: 10.3389/fnins.2018.00171 29615859 PMC5869197

[2] Karmali F, Goodworth AD, Valko Y, Leeder T, Peterka RJ, Merfeld DM. The role of vestibular cues in postural sway. J Neurophysiol. 2021;125(2):672–86. doi: 10.1152/jn.00168.2020 33502934 PMC7948142

[3] Dewan BM, James CR, Kumar NA, Burgess N, Zupancic S, Sawyer SF. Adaptation in motor strategies for postural control associated with sensory reweighting. Hum Mov Sci. 2023;89:103098. doi: 10.1016/j.humov.2023.103098 37167903

[4] Missen KJ, Carpenter MG, Assländer L. Velocity dependence of sensory reweighting in human balance control. Journal of Neurophysiology. 2024;132(2):454–60. doi: 10.1152/jn.00075.2024 38958285

[5] Ebrahimi A-A, Movallali G, Jamshidi A-A, Rahgozar M, Haghgoo HA. Postural control in deaf children. Acta Med Iran. 2017:115–22 28282708

[6] Melo RS, Lemos A, Raposo MCF, Monteiro MG, Lambertz D, Ferraz KM. Repercussions of the Degrees of Hearing Loss and Vestibular Dysfunction on the Static Balance of Children With Sensorineural Hearing Loss. Physical Therapy. 2021;101(10):pzab177. doi: 10.1093/ptj/pzab177 34270771

[7] Chisari D, Vitkovic J, Clark R, Rance G. Vestibular function and balance performance in children with sensorineural hearing loss. International Journal of Audiology. 2024;63(11):875–83. 38071612 10.1080/14992027.2023.2281878

[8] Zarei H, Norasteh AA, Lieberman LJ, Ertel MW, Brian A. Balance control in individuals with hearing impairment: A systematic review and meta-analysis. Audiol Neurootol. 2024;29(1):30–48. doi: 10.1159/000531428 37557094

[9] An M-h, Yi C-h, Jeon H-s, Park S-y. Age-related changes of single-limb standing balance in children with and without deafness. International journal of pediatric otorhinolaryngology. 2009;73(11):1539–44. 19720404 10.1016/j.ijporl.2009.07.020

[10] Wolter NE, Gordon KA, Campos J, Madrigal LDV, Papsin BC, Cushing SL. Impact of the sensory environment on balance in children with bilateral cochleovestibular loss. Hearing Research. 2021;400:108134. doi: 10.1016/j.heares.2020.108134 33310565

[11] Hazen M, Cushing SL. Implications of concurrent vestibular dysfunction in pediatric hearing loss. Current Otorhinolaryngology Reports. 2020;8:267–75.

[12] Walicka-Cupryś K, Przygoda Ł, Czenczek E, Truszczyńska A, Drzał-Grabiec J, Zbigniew T, Tarnowski A. Balance assessment in hearing-impaired children. Research in developmental disabilities. 2014;35(11):2728–34. 25077831 10.1016/j.ridd.2014.07.008

[13] Seyedi M, Seidi F, Minoonejad H. An Investigation of the efficiency of sensory systems involved in postural control in deaf athletes and non-athletes. Journal of Exercise Science and Medicine. 2015;7(1):111–27.

[14] Azadian E, Majlesi M, Saberifar S. Linear and Non-Linear Changes of Center of Pressure due to Vestibular System Disorders: Comparison of Balance and Gait in Hearing and Sensorineural Deaf Children. Pajouhan Scientific Journal. 2023;21(3):175–85.

[15] Daneshmandi H, Norasteh AA, Zarei H. Balance in the blind: A systematic review. Physical Treatments-Specific Physical Therapy Journal. 2021;11(1):1–12.

[16] Alghadir AH, Alotaibi AZ, Iqbal ZA. Postural stability in people with visual impairment. Brain and behavior. 2019;9(11):e01436. 10.1002/brb3.1436 PMC6851802 31578824

[17] Tomomitsu MS, Alonso AC, Morimoto E, Bobbio TG, Greve J. Static and dynamic postural control in low-vision and normal-vision adults. Clinics. 2013;68:517–21. 23778351 10.6061/clinics/2013(04)13 PMC3634964

[18] Zarei H, Norasteh AA, Lieberman LJ, Ertel MW, Brian A. Balance Control in Individuals With Visual Impairment: A Systematic Review and Meta-Analysis. Motor Control. 2023;27(4):677–704. doi: 10.1123/mc.2022-0127 37433525

[19] Urbaniak-Olejnik M, Loba W, Stieler O, Komar D, Majewska A, Marcinkowska-Gapińska A, Hojan-Jezierska D. Body balance analysis in the visually impaired individuals aged 18–24 years. International Journal of Environmental Research and Public Health. 2022;19(21):14383. doi: 10.3390/ijerph192114383 36361259 PMC9654500

[20] Müürsepp I, Arjokesse R, Ereline J, Pääsuke M, Gapeyeva H. Impact of visual impairment on static and dynamic postural control and habitual physical activity in children aged 10–16 years. British Journal of Visual Impairment. 2018;36(3):227–37.

[21] Schmid M, Nardone A, De Nunzio AM, Schmid M, Schieppati M. Equilibrium during static and dynamic tasks in blind subjects: no evidence of cross-modal plasticity. Brain. 2007;130(8):2097–107. 17611240 10.1093/brain/awm157

[22] Häkkinen A, Holopainen E, Kautiainen H, Sillanpää E, Häkkinen K. Neuromuscular function and balance of prepubertal and pubertal blind and sighted boys. Acta Paediatr. 2006;95(10):1277–83. doi: 10.1080/08035250600573144 16982502

[23] Walicka-Cupryś K, Rachwał M, Guzik A, Piwoński P. Body balance of children and youths with visual impairment (pilot study). Int J Environ Res Public Health. 2022;19(17):11095. doi: 10.3390/ijerph191711095 36078810 PMC9518479

[24] Farsi A, Abdoli B, Najafi K. Dual task effects in sensory systems interference condition on blind and sighted persons balance. Motor Behavior. 2014;6(15):15–28.

[25] Purpura G, Del Magro EF, Caputo R, Cioni G, Tinelli F. Visuo-haptic transfer for object recognition in children with peripheral visual impairment. Vision Research. 2021;178:12–7. doi: 10.1016/j.visres.2020.06.008 33070030

[26] Mohammadi F, Bayati M, Abbasi H, Allafan N. Better functioning of the somatosensory system in postural control of blind athletes compared to non-athletes. Scientific Journal of Rehabilitation Medicine (SJRM). 2019;8(3):179–87.

[27] Suarez H, Angeli S, Suarez A, Rosales B, Carrera X, Alonso R. Balance sensory organization in children with profound hearing loss and cochlear implants. Int J Pediatr Otorhinolaryngol. 2007;71(4):629–37. doi: 10.1016/j.ijporl.2006.12.014 17275927

[28] Peterka RJ. Sensory integration for human balance control. Handb Clin Neurol. 2018;159:27–42. doi: 10.1016/B978-0-444-63916-5.00002-1 30482320

[29] Zhang L, Feldman AG, Levin MF. Vestibular and corticospinal control of human body orientation in the gravitational field. Journal of Neurophysiology. 2018;120(6):3026–41. doi: 10.1152/jn.00483.2018 30207862 PMC6337031

[30] Negahban H, Nassadj G. Effect of hearing aids on static balance function in elderly with hearing loss. Gait & Posture. 2017;58:126–9. 28772132 10.1016/j.gaitpost.2017.07.112

[31] Gribble PA, Hertel J. Considerations for Normalizing Measures of the Star Excursion Balance Test. Measurement in Physical Education and Exercise Science. 2003;7(2):89–100. doi: 10.1207/s15327841mpee0702_3

[32] Moghaddam MB, Aghdam FB, Jafarabadi MA, Allahverdipour H, Nikookheslat SD, Safarpour S. The Iranian Version of International Physical Activity Questionnaire (IPAQ) in Iran: content and construct validity, factor structure, internal consistency and stability. World Appl Sci J. 2012;18(8):1073–80.

[33] Lohman TG, Roche AF, Martorell R. Anthropometric standardization reference manual. Champaign, IL: Human Kinetics. 1988.

[34] Lin D, Seol H, Nussbaum MA, Madigan ML. Reliability of COP-based postural sway measures and age-related differences. Gait & posture. 2008;28(2):337–42. 18316191 10.1016/j.gaitpost.2008.01.005

[35] Hadian MR, Negahban H, Talebian S, Salavati M, Jafari AH, Sanjari MA, et al. Reliability of center of pressure measures of postural stability in patients with unilateral anterior cruciate ligament injury. J Appl Sci. 2008;8(17):3019–25.

[36] Salavati M, Hadian MR, Mazaheri M, Negahban H, Ebrahimi I, Talebian S, et al. Test–retest reliabty of center of pressure measures of postural stability during quiet standing in a group with musculoskeletal disorders consisting of low back pain, anterior cruciate ligament injury and functional ankle instability. Gait & posture. 2009;29(3):460–4. 19167891 10.1016/j.gaitpost.2008.11.016

[37] Grove CR, Whitney SL, Hetzel SJ, Heiderscheit BC, Pyle GM. Validation of a next-generation sensory organization test in adults with and without vestibular dysfunction. J Vestib Res. 2021;31(1):33–45. doi: 10.3233/VES-200040 33325418 PMC8202570

[38] McNerney KM, Coad ML, Burkard RF. Learning effects and the sensory organization test: influence of a unilateral peripheral vestibular impairment. Am J Audiol. 2018;27(4):539–46. doi: 10.1044/2018_AJA-17-0067 30357271

[39] Brandt T, Krafczyk S, Malsbenden I. Postural imbalance with head extension: improvement by training as a model for ataxia therapy. Ann N Y Acad Sci. 1981;374:636–49. doi: 10.1111/j.1749-6632.1981.tb30907.x 6978651

[40] Patel M, Fransson P-A, Johansson R, Magnusson M. Foam posturography: standing on foam is not equivalent to standing with decreased rapidly adapting mechanoreceptive sensation. Exp Brain Res. 2011;208:519–27. doi: 10.1007/s00221-010-2498-6 21120458

[41] Zarei H, Norasteh AA. The effect of 8 weeks proprioception training without visual input on single-limb standing balance time in deaf students: A randomized controlled trial. J Bodyw Mov Ther. 2020;24(2):63–8. doi: 10.1016/j.jbmt.2019.09.002 32507154

[42] Field A. Discovering statistics using IBM SPSS statistics. Sage; 2013.

[43] Cohen J. Statistical power analysis for the behavioral sciences. Hillsdale, NJ: Lawrence Earlbaum Associates Publisher. 1988;8–16.

[44] Ade JD, Harley JA, Bradley PS. Physiological response, time-motion characteristics, and reproducibility of various speed-endurance drills in elite youth soccer players: small-sided games versus generic running. International Journal of Sports Physiology and Performance. 2014;9(3):471–9. doi: 10.1123/ijspp.2013-0390 24509482

[45] de Sá CdSC, Boffino CC, Ramos RT, Tanaka C. Development of postural control and maturation of sensory systems in children of different ages a cross-sectional study. Brazilian journal of physical therapy. 2018;22(1):70–6. 29239806 10.1016/j.bjpt.2017.10.006 PMC5816079

[46] Rajendran V, Roy FG, Jeevanantham D. Postural control, motor skills, and health-related quality of life in children with hearing impairment: a systematic review. Eur Arch Otorhinolaryngol. 2012;269(4):1063–71. doi: 10.1007/s00405-011-1815-4 22057941

[47] Parreira RB, Grecco LAC, Oliveira CS. Postural control in blind individuals: A systematic review. Gait Posture. 2017;57:161–7. doi: 10.1016/j.gaitpost.2017.06.008 28641161

[48] Dijkstra BW, Bekkers EM, Gilat M, de Rond V, Hardwick RM, Nieuwboer A. Functional neuroimaging of human postural control: A systematic review with meta-analysis. Neuroscience & Biobehavioral Reviews. 2020;115:351–62. 32407735 10.1016/j.neubiorev.2020.04.028

[49] Gerber ED, Huang C-K, Moon S, Devos H, Luchies CW. Sensory reweighting of postural control requires distinct rambling and trembling sway adaptations. Gait Posture. 2024;112:16–21. doi: 10.1016/j.gaitpost.2024.04.028 38723391

[50] Atilgan O. Relationships between perceptual-motor skills and postural balance in nine years old boys. Educ Res Rev. 2012;7(24):517

[51] Gao Z, Wen X, Fu Y, Lee JE, Zeng N. Motor skill competence matters in promoting physical activity and health. BioMed Research International. 2021; 2021:1–5. 35028311 10.1155/2021/9786368 PMC8748756

[52] Chen H, Housner L. The Relationship among Health-Related Fitness, Motor Skills Performance, and Physical Activity in Middle School Students. Asian Journal of Exercise & Sports Science. 2013;10(2).

[53] Parreira RB, Silva JG da , Nascimento MdM, Galli M, Oliveira CS. Effects of the interference of sensory systems on postural control in congenitally blind subjects. J Mot Behav. 2023;55(3):237–44. doi: 10.1080/00222895.2022.2156453 36572416

[54] Castaldi E, Lunghi C, Morrone MC. Neuroplasticity in adult human visual cortex. Neurosci Biobehav Rev. 2020;112:542–52. doi: 10.1016/j.neubiorev.2020.02.028 32092315

[55] Beauchamp MS, Oswalt D, Sun P, Foster BL, Magnotti JF, Niketeghad S, et al. Dynamic stimulation of visual cortex produces form vision in sighted and blind humans. Cell. 2020;181(4):774–83. e5. 32413298 10.1016/j.cell.2020.04.033 PMC7331799

[56] Oldenburg IA, Hendricks WD, Handy G, Shamardani K, Bounds HA, Doiron B, Adesnik H. The logic of recurrent circuits in the primary visual cortex. Nature neuroscience. 2024;27(1):137–47. 38172437 10.1038/s41593-023-01510-5 PMC10774145

[57] Mowad TG, Willett AE, Mahmoudian M, Lipin M, Heinecke A, Maguire AM, et al. Compensatory cross-modal plasticity persists after sight restoration. Front Neurosci. 2020;14:291. doi: 10.3389/fnins.2020.00291 32477041 PMC7235304

[58] Ribeiro F, Oliveira J. Aging effects on joint proprioception: the role of physical activity in proprioception preservation. European Review of Aging and Physical Activity. 2007;4:71–6.

[59] Héroux ME, Butler AA, Robertson LS, Fisher G, Gandevia SC. Proprioception: a new look at an old concept. American Physiological Society Rockville, MD; 2022. 811–4. 10.1152/japplphysiol.00809.2021 35142561

[60] Derlich M, Kręcisz K, Kuczyński M. Attention demand and postural control in children with hearing deficit. Research in Developmental Disabilities. 2011;32(5):1808–13. 21482067 10.1016/j.ridd.2011.03.009

[61] Peterson ML, Christou E, Rosengren KS. Children achieve adult-like sensory integration during stance at 12-years-old. Gait & posture. 2006;23(4):455–63. 16002294 10.1016/j.gaitpost.2005.05.003

[62] Goldberg JM. Vestibular inputs: The vestibular system. Neuroscience in the 21st Century: From Basic to Clinical. Springer; 2022. p. 1291–338.

[63] Norasteh AA, Zarei H, Mollazadeh M. Organization of Sensory Systems in Postural Control of Children: A Review of Literature. The Scientific Journal of Rehabilitation Medicine. 2020; 9(4):298–307.

[64] Ebrahimi AA, Jamshidi AA, Movallali G, Rahgozar M, Haghgoo HA. The effect of vestibular rehabilitation therapy program on sensory organization of deaf children with bilateral vestibular dysfunction. Acta Medica Iranica. 2017:683–9. 29307157

[65] Coto J, Alvarez CL, Cejas I, Colbert BM, Levin BE, Huppert J, et al. Peripheral vestibular system: Age-related vestibular loss and associated deficits. Journal of Otology. 2021;16(4):258–65. 34548873 10.1016/j.joto.2021.06.001 PMC8438634

[66] Rine RM. Growing evidence for balance and vestibular problems in children. Audiological Medicine. 2009;7(3):138–42. doi: 10.1080/16513860903181447

[67] Rogge A-K, Hötting K, Nagel V, Zech A, Hölig C, Röder B. Improved balance performance accompanied by structural plasticity in blind adults after training. Neuropsychologia. 2019;129:318–30. doi: 10.1016/j.neuropsychologia.2019.04.005 31004689

[68] Helmich I, Gemmerich R. Neuronal Control of Posture in Blind Individuals. Brain Topography. 2024:1–13. 10.1007/s10548-024-01041-7 PMC11393032 38491332

