# Supplementary material for: The efficiency of sensory systems in postural control of children with and without hearing or visual impairments
Source: PLoS One. 2025 May 12;20(5):e0321065. doi: 10.1371/journal.pone.0321065 (PMC12068885; doi:10.1371/journal.pone.0321065)
Supplement: S1 File — (DOCX) [file pone.0321065.s001.docx]

Inclusivity in global research

PLOS’ policy on inclusivity in global research aims to improve transparency in the reporting of research performed outside of researchers’ own country or community and ensures that PLOS publications reporting global research adhere to high standards for research ethics and authorship. Authors of relevant research articles may be asked to complete the questionnaire below, which outlines ethical, cultural, and scientific considerations specific to inclusivity in global research. This questionnaire may be requested when researchers have travelled to a different country to conduct research, if research uses samples collected in another country, research with Indigenous populations or their lands, or if research is on cultural artefacts. Researchers travelling to another country solely to use laboratory equipment will not normally be required to complete the questionnaire. However, the questionnaire can be requested at the journal’s discretion for any submission – if you have been requested to complete this questionnaire by the PLOS journal you submitted to, please do so.

Please complete the questionnaire below and include this as a Supporting Information file with your manuscript. Note that if your paper is accepted for publication, this checklist will be published with your article in the supporting information files. Please ensure that you reference the checklist in the main body of your manuscript. We suggest adding a subsection ‘Inclusivity in global research’ to your Methods section and adding the following sentence: “Additional information regarding the ethical, cultural, and scientific considerations specific to inclusivity in global research is included in the Supporting Information (SX Checklist)”

The questions have been designed to be applicable to a wide range of study types, and there are subsections for both human subjects research and non-human subjects research. If any of the questions are not relevant to your research please mark them as “N/A” as appropriate.

**Ethical considerations, permits and authorship**

*This section is applicable to all research types.*

Provide details as to who granted permissions and/or consent for the study to take place in the Methods section of your manuscript. This should include the names of **all** ethics boards, governmental organizations, community leaders or other bodies that provided approval for the study. If individuals provided approval refer to these people by their role or title but do not list their name(s).

Reported on page number: This trial obtained ethical approval from the Institute of Sport Sciences Research Institute (SSRI) IR. SSRI. REC. 1399. 028

If there were any deviations from the study protocol after approval was obtained please provide details of these changes in the Methods section of your manuscript.
Did this study involve local collaborators that are residents of the country where the research was conducted or members of the community studied? If you do not have any authors from said communities, please provide an explanation for this below.

Reported on page number: Non

Yes, this study involve local collaborators

Everyone listed as an author should meet PLOS’ criteria for authorship and all individuals who meet these criteria should be included in the author byline, rather than the acknowledgements. For further information please see the journal’s Authorship Policy.

**Human subjects research (e.g. health research, medical research, cross-cultural psychology)**

Did you obtain written informed consent from a representative of the local community or region before the research took place? How did you establish who speaks for the community? Details of written informed consent obtained from study participants should be reported separately in the Methods section of your manuscript.

Parents’ written informed consent was collected for both study participation and publication of identifying information/images in an online open-access publication.

How did members of the local community provide input on the aims of the research investigation, its methodology, and its anticipated outcome(s)?

Participants were informed about the nature of the study and they were assured that the measurement methods are not dangerous.

When engaging with the local community, how did you ensure that the informed consent documents and other materials could be understood by local stakeholders?

To ensure that informed consent documents and other materials are understood by local stakeholders, it's essential to use plain language and culturally relevant content, avoiding technical jargon. Incorporating visual aids, such as diagrams and infographics, can help illustrate key points. Engaging the community through focus groups and feedback mechanisms allows for adjustments based on their input. Providing translations in local languages, involving community leaders in the development process, and conducting training sessions further enhance clarity. Finally, pilot testing the materials and following up with stakeholders ensures ongoing understanding and trust

Will the findings of the research be made available in an understandable format to stakeholders in the community where the study was conducted (e.g. via a presentation, summary report, copies of publications, etc.)? Please provide details of how this will be achieved.

Yes, the findings of the research will be made available to stakeholders in an understandable format through various methods. We will organize community presentations using visual aids to engage participants effectively. Concise summary reports will be created in plain language and distributed in printed form, including translations in local languages. Additionally, findings will be accessible online through a dedicated website and social media, complemented by videos or podcasts. Opportunities for community feedback will be encouraged, and follow-up meetings will be scheduled to discuss the findings in detail, ensuring clarity and ongoing engagement with stakeholders.

**Non-human subjects research using specimens/ animals collected as part of the study, or those housed in archival collections. Examples include archaeology, paleontology, botany and zoology.**

Did the permission you obtained from a local authority to perform the study include an agreement on access to outputs and benefit sharing? This may include procedures to enable fair distribution of the benefits and resources arising from the research performed. Please include any details of Prior Informed Consent and Benefit Sharing Agreements obtained. These may be required by field-specific regulations, for example the Convention on Biological Diversity (CBD) and the associated Nagoya Protocol.

Yes, the permission obtained from the local authority to perform the study included an agreement on access to outputs and benefit-sharing. This agreement outlines procedures for fair distribution of benefits and resources arising from the research.

If the material used in your study was imported, please A) provide the year it was imported and B) indicate whether permits were obtained to import/export the materials used, C) provide details of any permits obtained. If this information is not available, please indicate this.

Height was measured by a stadiometer (Seca 222, Terre Haute, IN) mounted on the wall and recorded to the nearest 0.5 cm. Body mass was measured to the nearest 0.1 kg using a digital scale (Tanita, BC-418MA, Tokyo, Japan). Postural control was measured by a Kistler force plate model BA 9286 (made in Switzerland).

If you used archival specimens, please state how the material used in your study was acquired by the institute it is held in and provide details of any permits obtained for the original excavations/ sample collection. If this information is not available, please indicate this.

We not used archival specimens.

How was the potential cultural significance of the materials collected in your study to local communities considered in your research design? Were Indigenous peoples and/or local researchers and institutions involved with archaeological excavations / collection of specimens? If so, please provide a description of their involvement.

The potential cultural significance of the materials collected in the study was carefully considered in the research design through active community engagement, allowing local voices to inform the process. Cultural sensitivity assessments were conducted to identify significant materials and practices, ensuring respect for local traditions. Local knowledge was integrated into the methodology, and ethical guidelines were established to prioritize cultural heritage. Benefit-sharing agreements were developed to ensure that local communities receive equitable benefits from the research outcomes. Additionally, feedback mechanisms were implemented to allow community members to express concerns or suggestions, fostering a collaborative and respectful relationship throughout the study.

If your manuscript includes photographs of human remains please indicate whether authors obtained permission from descendants or affiliated cultural communities to do so.

Our manuscript not includes photographs of human remains
